# A Qualitative Exploration of Addiction Disclosure and Stigma among Faculty Members in a Canadian University Context

**DOI:** 10.3390/ijerph18147274

**Published:** 2021-07-07

**Authors:** Victoria F. Burns, Christine A. Walsh, Jacqueline Smith

**Affiliations:** Faculty of Social Work, University of Calgary, Calgary, AB T2E 1M2, Canada; cwalsh@ucalgary.ca (C.A.W.); jacqueline.smith1@ucalgary.ca (J.S.)

**Keywords:** addiction, alcohol, stigma, disclosure, university faculty, help-seeking, workplace

## Abstract

Addiction is one of the most stigmatized public health issues, which serves to silence individuals who need help. Despite emerging global interest in workplace mental health and addiction, scholarship examining addiction among university faculty members (FMs) is lacking, particularly in a Canadian context. Using a Communication Privacy Management (CPM) framework and semi-structured interviews with key informants (deans and campus mental health professionals), this qualitative study aimed to answer the following research questions: (1) What is the experience of key informants who encounter FM addiction? (2) How may addiction stigma affect FM disclosure and help-seeking? and (3) What may help reduce addiction stigma for FMs? Thematic analysis was used to identify three main themes: (1) Disclosure was rare, and most often involved alcohol; (2) Addiction stigma and non-disclosure were reported to be affected by university alcohol and productivity cultures, faculty type, and gender; (3) Reducing addiction stigma may involve peer support, vulnerable leadership (e.g., openly sharing addiction-recovery stories), and non-discriminatory protective policies. This study offers novel insights into how addiction stigma may operate for FMs in relation to university-specific norms (e.g., drinking and productivity culture), and outlines some recommendations for creating more recovery-friendly campuses.

## 1. Introduction

### 1.1. Background

This exploratory qualitative study is inspired by my (first author) complicated experience disclosing an addiction-recovery identity in an academic context. Specifically, in a recent article [1], I expose several risks, benefits, and paradoxes of disclosing an alcohol addiction-recovery identity in a Western academic environment, where drinking, often to excess, is not only accepted but encouraged [2].

Both as a sober PhD student and junior faculty member (FM), academia was one of the last places where I felt safe disclosing. Warned by more senior academics that disclosure may negatively affect my reputation and job prospects, for the first five years of my recovery, I told very few people. As an addiction researcher and social work educator, the constant “calculus of disclosure” [3] (p. 688) was not only exhausting, but it was negatively affecting my teaching, research, and the overall quality of my recovery. Through various personal encounters with other academics with addiction, I came to understand that I was not alone in experiencing the stigma-fueled burden of negotiating what Petronio [4] refers to as privacy boundaries. After years of carefully weighing the disclosure risks and benefits in different contexts, I reached a point where I could no longer manage the stress of living a double life in recovery. When I explained to my dean that my fear of disclosure was negatively affecting my work and recovery, I was fortunately met with understanding and compassion. He informed me that in his 26-year career, while many academics disclosed openly about physical and other mental health issues (e.g., depression, anxiety), I was only the second FM who disclosed about addiction.

On the one hand, the invisibility he reported was not surprising given the extent of public and self-stigma associated with addiction, and its shame-fueled silencing effect [5]. On the other hand, considering one in five Canadians experience addiction in any given year [6], the extent of his reported silence is somewhat alarming. With over 47,000 full-time academic teaching staff at public universities in Canada [7], this could translate into more than 10,000 FMs who are experiencing addiction across the country. Based on statistics alone, the number of FMs falling somewhere along the addiction-recovery spectrum at my university could be in the hundreds—but no one was talking about it, and I felt very isolated in my experience. The positive experience I had with my dean led me to further question whether and how other deans and/or campus mental health professionals were encountering FMs with addiction? What were their experiences? What kind of help was being provided to FMs? What were the gaps?

In recognizing that research focusing on FMs direct experience of disclosing addiction is sorely needed [8,9], as a first step, this study considers the perspective of key informants (deans and campus mental health professionals) at one large research-intensive university in Western Canada. Specifically, using a Communications Privacy Management (CMP) Theory framework, this study advances the literature by exploring key informants’ encounters with FM addiction disclosure, the possible role of stigma in FM disclosure, and recommendations that may help reduce addiction stigma on campus for FMs.

Definitional clarification: This study is centering on addiction, and not substance use in general. Addiction is defined as the “problematic use of a substance, with harms ranging from mild (e.g., feeling hungover, being late for work) to severe (e.g., homelessness, disease)” [10] (n.p.). Substance use includes licit drugs (e.g., alcohol, prescription drugs, cannabis (cannabis was legalized across Canada on 19 June 2018), and illicit drugs (e.g., cocaine, methamphetamine, opioids). We also recognize that addiction-recovery is a process that includes periods of remission and relapse [11].

### 1.2. Extent of the Problem: Addiction Estimates among Faculty Members

The relationship between problematic substance use and lost productivity is well documented in the workplace literature [12]. While no specific national data on FMs is available, in any given week, at least 500,000 employed Canadians took time off work because of mental health issues, including addiction [13]. Alcohol alone accounts for $14.6 billion direct (e.g., hospital) and indirect costs (e.g., lost productivity) in Canada every year [14]. Large research-intensive Canadian universities have independently noted similar trends related to increased workplace stress and longer mental health–related disability leaves [15,16,17].

Workplace stress and norms (e.g., drinking culture) are associated with a higher incidence of problematic substance use [18]. Further, isolated work environments, low levels of supervision, and high mobility are also risk factors for problematic substance use [18], all of which are characteristics of academic work. Considering that workplace stress and burnout are prominent among academics, and that university campuses are known to be drug promoting environments [19,20,21], the prevalence of addiction for FMs may be higher than national estimates (i.e., one in five).

Despite high rates of addiction, a minority of people struggling with addiction seek help, often because of stigma [5]. For instance, USA-based data estimate that only 10.8% of individuals who met criteria for a substance use disorder (SUD) sought or received help [22].While rates of FM addiction may be buffered by education, higher socioeconomic status, individuals with higher education levels may be less likely to view themselves as needing help, thus creating a significant barrier to help-seeking when a problem with substance use arises [23]. Moreover, even when mental health resources are available in the workplace, most employees are deterred from seeking help because of stigma [5].

## 2. Guiding Concepts and Theoretical Framework

### 2.1. Addiction Stigma and Disclosure

According to Goffman [24], stigma occurs in a social context where a person is understood by others to have undesired differentness, leading to “othering” and a sense of self deemed as abnormal, deviant, flawed, or spoiled. Link and Phelan [25] extend Goffman’s classic definition, suggesting that stigma is amplified by power dynamics, which as Avery and Avery [26] suggest, affect perceived and enacted discrimination across different life domains, including the workplace [27].

Avery and Avery [26] (p. 2) define addiction stigma as “negative attitudes towards those suffering from substance use disorders that one, arise on account of the substance use disorder itself, and two, are likely to impact physical, psychological, social, or professional well being.” The public stigma of addiction can be attributed to common stereotypes, including dangerousness, criminality, sin, incompetence, hopelessness, no job potential, in denial, weak in character, and sick [9].

Research has found that addiction is more heavily stigmatized than other mental health issues (e.g., schizophrenia, severe depression, eating disorders) [5,28]. For example, a review of 17 population studies examining the stigma of alcoholism compared to other mental, medical, or social conditions (e.g., schizophrenia, severe depression, eating disorders, dementia, paralysis) found that people with alcohol dependence are viewed as much more responsible for their behaviors, which reinforces exclusion and discrimination [28].

When prejudices are internalized, people with addiction experience self-stigma, which is characterized by negative feelings and perceptions of self [29]. Further, structural stigma occurs when policies and procedures discriminate against people with addictions [30]. Compounding factors affecting addiction stigma include type of substance (e.g., it is more acceptable to disclose legal drug use [e.g., alcohol] than illegal drug use [31]). Individual characteristics also affect the degree of stigma experienced (e.g., if the person belongs to another stigmatized group based on race, class, gender, etc.) [32,33].

The concept of disclosure is inextricably tied to stigma, as it implies a need for concealment or a secret life [34]. Disclosure or self-disclosure refers to the process of communicating information about oneself verbally to another person [34]. Non-disclosure of a stigmatized identity in the workplace [35] can have negative consequences, including feelings of non-belonging, psychological distress, strained social interactions, and heightened risk for disease [36,37]. However, findings regarding disclosing stigmatized identities (e.g., LGBTQ+ identity, mental and physical illness) in the workplace are contradictory. While some studies have found that disclosure increases experiences of discrimination [38], others report that disclosing a stigmatized identity leads to better health outcomes and overall job satisfaction [35]. Overall, although workplace research has explored the risks and benefits of disclosing stigmatized identities in the workplace [35], little is known about the how addiction stigma may affect disclosure decisions for FMs with addiction in a Western academic context.

### 2.2. Communication Privacy Management Theory

Communication Privacy Management (CPM) Theory [4] is a useful framework for understanding university key informant’s experiences of FM addiction disclosure. CPM posits that people engage in a risk-benefit analysis to determine whether, when, and how to disclose. The predicament of disclosure requires individuals to create metaphorical privacy boundaries, which can be permeable (e.g., disclosing in some contexts) and impermeable (e.g., not disclosing). The disclosure risk and benefit assessment criteria are affected by cultural norms and expectations, such as university culture.

CPM has been a useful framework to examine how individuals with varied stigmatized identities manage disclosure and privacy. For example, Yep [39] explored disclosure decisions amongst individuals with HIV/AIDS finding that if participants anticipated a supportive response, they would disclose. Romo and colleagues have conducted extensive research on the dynamics and motivations of communicating a non-drinking identity in college student and employed populations [21,40,41,42]. For instance, using a CPM lens, Romo [21] interviewed non-drinking college students and found that the students concealed their non-drinking identity (erected an impermeable boundary) and engaged in stigma management tactics (e.g., always holding a cup at parties) to fit in. Building on the college study, Romo and colleagues [40] interviewed non-drinking professionals, finding that abstainers (including but not exclusively former problem drinkers) viewed non-drinking as a deviant act, and deployed a variety of preventive and corrective facework strategies (including passing and humor). Narrowing the study population to former problem drinkers (“alcoholics”), Romo et al. [41] found that most of the participants erected impermeable boundaries [4] around their non-drinking status because the risks of career discrimination were too great. However, similar to the students who identified as being in recovery in Romo et al. [41], several of the former problem-drinking professionals chose to disclose when they felt that the possibility of building relationships, staying true to themselves, serving as a role model for others, and maintaining their own sobriety outweighed the potential risks.

Overall, while research has focused on disclosure dialectics from the perspective of individuals with stigmatized identities, including non-drinkers in university and workplace contexts, scant research has focused on disclosure encounters with FMs with addiction. Redressing this gap in the literature, using a CPM framework and semi-structured interviews with key informants (10 deans and 6 campus mental health professionals (CMHPs), this exploratory qualitative study aimed to answer the following research questions:

1. What is the experience of deans and CMHPs who encounter FM addiction?
2. How may addiction stigma affect FM disclosure and help-seeking?
3. What may help reduce addiction stigma for FMs?

By capturing key informants’ perspectives, this study offers novel insights into how addiction stigma may operate in academia and outlines some recommendations and next steps that may help reduce addiction stigma for FMs.

## 3. Materials and Methods

### 3.1. Study Design and Sample

An exploratory qualitative design was used, where the aim is not to generalize but to provide a rich, nuanced understanding of a particular human experience or phenomenon [43,44]. Qualitative research is useful to begin exploring an area of study that has received little attention [44], which in the case in FMs addiction disclosure.

Recruitment: Sixteen faculty deans and eight CMHPs from one Western Canadian university were purposefully sampled through existing community connections or the university website. All identified key informants were sent a detailed letter of invitation via email. If the individual agreed to participate in the study, an interview was scheduled at a mutually convenient time and place. All were required to be in their role for at least one year. Six of the deans and two CMHPs declined the invitation to be interviewed, the most common reason being that they felt they would not be able to properly address the topic.

Sixteen in-depth interviews were conducted privately with 10 deans and 6 CMHPs (staff wellness, support staff, counsellors, and human resources roles). Interviews lasted from 30 to 60 min, averaging 40 min in length. A semi-structured approach was used to guide the discussion (included below). The interviews were conducted by one of the three authors. All interviews were done in person, except for one via telephone. 

The interview guide covered three main discussion areas: (1) understanding encounters with FM addiction; (2) perceptions of the risk of FM disclosure and help-seeking; and (3) recommendations for additional supports (see Table 1).

### 3.2. Ethics Approval

Ethics approval was obtained from the University Institutional Review Board, and written informed consent was provided by all key informants. The consent form outlined the process for ensuring confidentiality and protecting the right of informants to withdraw at any time during the study. Transcripts were carefully reviewed and de-identified to protect anonymity. No monetary compensation was provided to study participants.

### 3.3. Data Analysis

All interviews were audio-recorded, then transcribed verbatim by a research assistant using a word processor. Braun and Clarke’s [45] 5-Phase Framework for thematic analysis was used, which is a flexible method for identifying, analyzing, and reporting themes within data. 

Phase 1 of Braun and Clark’s method of analysis begins with data familiarization. The first author and the research assistant listened to the audio recordings and read all the transcripts (transcribed into a Microsoft Word file). For Phase 2 (generating initial codes), excerpts from transcripts were cut-and-pasted, using a Microsoft Excel spreadsheet, into broad preliminary descriptive categories that matched the areas probed in the interview guide: (1) types of drugs used (e.g., alcohol, cannabis, prescription); (2) negative consequences descriptors (e.g., erratic behaviors, driving under the influence); (3) mentions of university-specific culture affecting stigma (e.g., “competitive,” “pub nights,” “faculty club drinking,” “wine culture”), as well as words or phrases describing “vulnerability” (e.g., “getting real,” “authenticity,” “showing emotions”). (4) demographics identified as affecting addiction stigma and (non) disclosure (e.g., faculty type and gender); and (5) recommendations to reduce addiction stigma (e.g., peer support). 

In Phase 3, patterns were identified by the three authors, who discussed variations in the data, moving back and forth within and between cases. Of note, some of the patterns became evident based on frequency (e.g., direct disclosure was extremely rare), while others came directly from codes (e.g., faculty type and gender). This approach is consistent with thematic analysis, as outliers can be significant and become a theme, when they provide further insight into the research questions at hand [45]. 

In Phase 4, the three authors discussed and resolved discrepancies in how the codes would be organized into specific themes (e.g., “leading with vulnerability”). Finally, in Phase 5 (reviewing themes), three main themes were identified: (1) Disclosure was rare, and most often involved alcohol; (2) Addiction stigma and non-disclosure were reinforced by university-based alcohol and productivity cultures, faculty type, and gender; (3) Reducing addiction stigma may involve peer support, vulnerable leadership, and non-discriminatory protective policies.

## 4. Results

The following section describes the three themes with illustrative quotes drawn from the transcripts and labeled with the identification number. The deans represented 10 faculties ranging in size from approximately 30 to 400 faculty members. Most had more than 20 years of academic service (including professorial and senior leadership ranks). The CMHPs included staff wellness, counsellor, and human resources roles. Of the 16 key informants, five identified as women and the remainder identified as men. The majority were white. The team limited the demographic information collected, as the participants were recruited from a small pool of eligible interviewees, and we wanted to ensure their identities were protected as much as possible.

**Theme** **1.** *Addiction disclosure was rare, and most often involved alcohol*.

Although all the key informants expressed openness to being approached by FMs with a substance use problem, only two deans and one CMHP reported experiencing a direct disclosure and all three involved alcohol. As one dean expressed, this was surprising, given his length of time in service combined with awareness of the prevalence of addiction in broader society:

*I’ve had one conversation with an FM here at the university. That’s mind-boggling to me, and it suggests that we have a huge issue with stigma and a fear, perhaps, of going to others and having an open conversation about these issues and how to get support. For me that’s particularly troubling, because when we look at our broader community, we know that these are massive issues, and it would be naïve to think that they do not happen with the same intensity and rate that we see elsewhere in the community*.

(Dean 5)

The majority of the deans became aware of FM problematic substance use through a second party or other indirect means, including concerns expressed by students/colleagues, disruptions in work performance, erratic behavior, and legal consequences (e.g., driving under the influence), as the following two quotes illustrate:

*The person was becoming more disconnected within the department, and there were concerns from colleagues and the department head with respect to literally finding the person at the pub, in the afternoons, and not showing as much care around things like personal grooming*.

(Dean 2)

*He was a very smart person, uh … you know … [a] person of integrity and good work habits. However, then for periods he would just go—this isn’t a very precise term—but go off-kilter. His attendance would be spotty, sloppiness would be apparent in his work*.

(Dean 4)

Despite few FMs disclosing to their deans directly, when reflecting on their past experiences, several deans noticed signs of addiction, as this quote highlights:

*In retrospect, I’m just hypothesizing, but knowing about the alcohol issues that this individual had, it makes sense as to why he was never able to make morning meetings, was always late, and didn’t answer e-mail. He wasn’t just the absent-minded professor, there was actually something going on there*.

(Dean 7)

Another dean reported being aware of one FM who had a history of alcohol addiction but did not see it as an issue because the FM was performing and meeting targets:

*There was a prof who was an alcoholic. I only knew him professionally, but some of his colleagues were complaining that they could smell booze on him. Then I noticed his erratic behavior, and then he behaved very badly with some students,so I was mad at him. He ended up dying shortly after. But before the outburst with the students, he was doing his job, he handled a lot of teaching and students and generally was—for most of the time I’ve known him (which was at least 25 years)—I guess, he was a pretty high performing FM*.

(Dean 10)

In contrast, almost all the deans had direct encounters with FMs disclosing physical or mental health issues (e.g., cancer, bipolar disorder, depression, anxiety, grief). Several reported that the non-disclosure may be associated with the assumption that addiction is a personal failing and/or choice, and a weakness as the following three quotes highlight:

*My gut feeling is that [most people think] depression or anxiety-related disorders are not the fault of the individual. And they might assume, they could assume, that an addiction could be personality-related or just [someone] lacking self-control. Right? And so, I would say the risk of disclosing a substance abuse issue is probably higher than some of the medical, the well-known medical conditions that you might disclose from a mental health perspective*.

(Dean 3)

*There’s a stigma of shame with addiction because it’s perceived as negative coping, as opposed to something like a bipolar diagnosis, or anxiety. I think, by and large, they’re seen differently, even though we know it’s a chronic issue like any other chronic disease, but there’s the social stigma and shame attached. It doesn’t typically come up until there’s a negative consequence, right? I think that the attached or collateral shame around the negative consequence also contributes to it. I mean, so for instance if you had a diabetic who had a significant drop in their blood sugar and lost consciousness in the hallway, you know there’s not going to be the same perception of shame, as opposed to someone binge drinking and passing out in the classroom*.

(Dean 9)

*I think that there is more sympathy and understanding for physical impediments and conditions and ailments, things that you can see. For things that you can’t see, I think people tend to blame the victim, or blame the patient. People expect you to just “get over it.” If you can’t, you are viewed as weak*.

(Dean 6)

**Theme** **2.** *Addiction stigma and non-disclosure were reported to be affected by university alcohol and productivity cultures, faculty type, and gender*.

### 4.1. Alcohol Culture

Some of the key informants spoke to the pervasiveness of alcohol culture on campus. They described how drinking was not only normalized but encouraged among students, and also prevalent at faculty events (e.g., holiday parties, academic conferences). Although some key informants mentioned that alcohol culture has shifted away from the days of “boozy faculty lounges,” alcohol-centric activities such as “pub Fridays” are still common, as one dean suggested:

*There, there is a culture of alcohol in our faculty, it really isn’t nearly as prevalent as it was 20 or 30 years ago but there’s a few groups that I am aware of who head to the pub together on a Friday afternoon*.

(Dean 2)

Other key informants mentioned that alcohol culture could reinforce stigma, thus preventing disclosure, as the following quotes highlight:

*I think there are high stakes of disclosing a substance use disorder amongst faculty. That said, we live also in a culture where we make jokes about wine to help us at the end of the day, and you know, there’s certain parts of the culture I think that allow for it like, “Oh, let’s go get a drink!”, there’s a certain glam in the culture, where drinking socially is good and then we don’t kind of talk about the other stuff*.

(CMHP 5)

### 4.2. Productivity Culture

Several key informants perceived that the high level of non-disclosure related the high-pressure, competitive nature of academia, where any sign of vulnerability or weakness is strongly discouraged, as the following three quotes highlight:

*FMs are afraid to come out because of the fear of how it would be perceived. That an adult, somebody with a PhD, cannot handle day-to-day stress, that they would be considered weak, which may prevent [them from] getting a career opportunity*.

(Dean 1)

*I think that’s a big part of non-disclosure; it’s recognition, it’s competitiveness, its reputation*.

(Dean 7)

*FMs may not want to disclose addiction because [doing so] is voluntarily disclosing a potential weakness that, in a competitive world, could be a disadvantage. And it starts to color how people see your performance*.

(Dean 6)

Along similar lines, a CMHP pointed out that the culture of academia and specifically the emphasis on productivity and high achievement tends to mask potential issues related to addiction:

*I haven’t encountered a single FM with an addiction. Even though it’s 1 in 10 people, right? You know it’s because FMs are fairly high functioning. If you can get your papers out there and get grants then, you know, people don’t really care about the rest*.

(CMHP 5)

Further, some of the CMHPs identified that staff tend to reach out for help for mental health issues more often than FMs because they do not have the added pressures associated with status and reputation, as the following quote illustrates:

*I am aware of an FM with a substance issue but have not been approached by the person. It’s because of academic reputation. I hear more about people in supporting staff, but I don’t hear about FMs*.

(CMHP 3)

### 4.3. Faculty Type

Disclosure risks were also identified as being affected by the faculty the FM belonged to (e.g., professional). For instance, in the professional faculties (e.g., social work, medicine, nursing, and law), disclosing could lead to an FM being deemed unfit for practice and potentially losing his or her professional license. However, one dean described how being “fit to practice” in academia is not as clear as it is in a clinical setting where there is a duty to report problematic substance use:

*How intrusive can you be without being overly intrusive? If you try to intervene, they [the FM] might say, “Well it’s none of your business, and you’re harassing or bullying me,” or when you’re really coming out of a place of concern, and again [worry about the FM’s] fitness to practice, it’s more opaque in an academic institution than when you’re in [clinical] practice*.

(Dean 7)

Some of the deans reported specific risks associated with the nature of their work, such as risk factors associated with intoxication and being in a laboratory setting as Dean 7 pointed out:

*There’s another piece, though, that is really important to think about: there are a lot of things that university professors do that could kill you. Right? And so, when we compare it to, you know, if you’re human resources staff, if you come into work drunk, [at least] you’re not going to blow up the lab*.

### 4.4. Gender

One of the key informants reported that it was riskier for women to experience addiction and seek help than it was for their male colleagues, especially if they are pre-tenure, as the following quote highlights:

*There are different ways we describe male substance abuse versus female substance abuse. For women, everything is a strike against them when it comes to tenure, so they are ultra-careful and have to be more perfect than the men*.

(Dean 7)

**Theme** **3.** *Reducing stigma through peer support, vulnerable leadership, and non-discriminatory protective policies*.

### 4.5. Peer Support

All key informants highlighted the major gap in services for FMs, commenting that most target students. For instance, some of the key informants pointed out that there is a harm reduction support advisor specific for students, there used to be a student-led Alcoholics Anonymous (AA) group, and additional student peer-support groups led by the campus faith and spirituality center. 

Several key informants suggested that a promising way forward would be to offer university-based peer-support that is exclusive to academics as the following three quotes highlight:

*Peer support is certainly something on the student side that we’re keeping an eye on. I’ve talked about it for staff and faculty, but it’s not quite got that same sort of accessibility, I think, for staff in faculty, but it is something in terms of the Campus Mental Health Strategy that we’re starting to have conversations about*.

(CMHP 5)

*What’s missing is a peer model where somebody who’s been through addiction themselves offers their time, like a spiritual director would. People go to therapy, counselling, psychology, psychiatry, or they go to an addiction treatment center. I think peer counselling is what’s missing*.

(CMHP 4)

*I think it would be interesting if there were a recovery meeting, a 12-step program with a cohort of other academics. It would be doubly supportive because you’re working through your addiction, but you also have this shared knowledge of what the academic context is*.

(Dean 9)

### 4.6. Reducing Stigma through “Vulnerable Leadership”

A number of key informants suggested that addiction stigma could be reduced if a conversation about addiction amongst FM and staff started. Supporting this view, CMHP5 stated, “faculty need to start getting real about what is really going on, they need to start taking off the mask, and talking about addiction.” As some CMHPs alluded to, the conversation has to start “from the top” through what several referred to as “vulnerable leadership.” The prestige associated with being an FM was reported to affect non-disclosure, because as one CMHP expressed, it “doesn’t leave much room to make mistakes and be human.” Some pointed out that if FMs are going to feel safe disclosing, they need to make sure they are going to be met with compassion, and “not lose their job,” as another key informant suggested.

*The community becomes safer when those in more of an authoritative space lead with vulnerability*.

(CMHP 2)

Finally, to reduce addiction stigma for FMs, several key informants suggested normalizing addiction-recovery by having more campus community members (e.g., FMs, deans, administrators) share openly about their personal experiences, as has been done with other mental illnesses, as CMHP 1 suggests:

*People have conceptions of what mental illness is, and that conception doesn’t necessarily include addictions. I think we have to be a little more explicit on that. Maybe it’s bringing in people to talk about their experiences with alcohol or other substances. We had one FM speak openly about her anxiety and how she’s dealt with it—that got a lot of uptake, right? The video was viewed by thousands of our community members*.

### 4.7. Protective Policies

Several key informants suggested having policies and procedures in place so that FMs could safely discuss substance use concerns without consequences (e.g., without risk of losing or not getting tenure/promotions) and get adequate help. For instance, one of the CMHPs explained that not being reassured about their job security might serve as a major obstacle for FMs in disclosing and seeking help:

*FMs are known for what they do, but are we providing the resources to allow them to come forward without consequence?*

(CMHP 4)

Several also reported that if they did encounter an FM with a substance use problem, they would recommend the FM to employee assistance programs (EAP). However, they also elaborated that EAP is often inadequate to address addiction issues:

*I have my doubts about referrals to EAP for people who are seeking support for addictions. When a person reaches out for help and doesn’t get it, the person goes 15 steps backward in terms of ever asking for help again. We tell people we have an EAP program and give them a pamphlet that I find so simplistic and patronizing, like how (will this help?) It’s about facilitating that (request for support), not just about “here’s a card, here’s a card, here’s a card.”*

(CMHP 3)

## 5. Discussion

This qualitative study explored 10 dean and 6 CMHPs encounters with FM addiction, how addiction stigma may have affected FM (non) disclosure, and recommendations that may help reduce addiction stigma for FMs.

Using a CPM framework, this study makes a unique contribution to the literature as it provides insights into how alcohol culture, productivity culture, faculty type and gender may amplify disclosure risks/impermeable boundaries [4], thereby providing insights into the strikingly low number of FM disclosure reported by the key informants. Drawing on relevant literature, we discuss the identified risk criteria reported to affect stigma and FM disclosure, followed by an examination of possible benefits of disclosure.

### 5.1. Alcohol Culture

The interviews revealed that addiction disclosure was rare and most often involved alcohol. This finding is consistent with organizational literature suggesting that alcohol is the most frequently used drug by employed people [12]. Alcohol is also the most commonly used drug globally and being used at increasingly harmful rates [46]. For instance, in 2020, 78% of Canadians reported using alcohol in the past year, a quarter of whom (approximately 9.5 million) reporting heavy drinking (above low-risk guidelines) at least once a month [47].

While research has yet to focus on FMs direct experiences disclosing addiction or recovery, several studies have shown that college drinking culture leads non-drinkers to view abstinence as deviant and stigmatized, which affects their decisions to disclose [20,21,42]. Although the peer-pressure and temptations to drink may be different for FMs, given the length of time FMs are immersed into campus life (e.g., over the course of their student careers), it would be difficult not to be indoctrinated into the “abstinence-hostile collegiate environment” [48] to some degree. Additionally, Western workplaces are increasingly becoming alcogenic environments, where “the keg is becoming the new water cooler” [49] (para 1), which affects non-drinkers decisions to disclose, as they view abstinence as culturally deviant.

Study findings also revealed that key informants were being made aware of problematic drinking through negative consequences (e.g., drinking under the influence, erratic behavior in the classroom). This finding leads us to question the extent to which FMs were seeking help for addiction elsewhere. Having an impermeable privacy boundary [4] may reinforce denial and serve as a major barrier to seeking treatment and recovery for people with substance use disorders [42].

Therefore, in order to assist help-seeking and/or the maintenance of recovery for FMs who find themselves along the alcohol addiction-recovery continuum, efforts to change university drinking culture may be promising may forward to reduce addiction stigma. For instance, ensuring more ethical and responsible alcohol advertising on campuses [50], serving non-alcoholic alternatives, or establishing alcohol-free university events [9], may help reduce alcohol harms, normalize sobriety, reduce stigma and ultimately promote help-seeking for FMs. 

### 5.2. Productivity Culture

Several study participants reported that the highly competitive nature of academia created an additional barrier to disclosing, as “any sign of weakness” could negatively affect their reputations and careers. Supporting this view, it has been suggested that “research-intensive universities create cultures that demand high performance while promoting excellence and achievement, and also carry the risk of stress, stigma, and challenges to mental health” [17] (p. 1). Some participants also described how productivity culture could potentially mask addiction because as one key informant aptly expressed, “As long as targets are being met, nobody cares.”

Numerous participants identified that having more “vulnerable leadership” could help reduce addiction stigma thus help to promote FM disclosure and help-seeking. Several linked the concept of vulnerable leadership to encouraging the university community to share more openly about their addiction-recovery journeys. This finding aligns with other research suggesting that having access to sober role models, is an effective way to combat stigma and ultimately promote and sustain recovery [5]. It also aligns with the “bring your whole self to work” movement, based on the idea that having a more vulnerable, authentic workplace is effective in promoting improved health and wellness [51]. 

### 5.3. Gender

While the small sample of this study did not permit a thorough exploration of identity markers, one key informant pointed to the additional risks that disclosing an addiction identity would hold for female FMs. This finding is consistent with previous literature, suggesting that addiction is more heavily stigmatized for women than it is for men, which affects women’s ability to seek help [32]. Qualitative research has also found that compared to men, women experience more discrimination and denigrating attitudes (e.g., unfit mother, promiscuous) when they disclose a drug user identity [52]. More research exploring gender and addiction disclosure among FMs is needed.

### 5.4. Faculty Type

Faculty type was also identified as affecting disclosure risk. Some research has emphasized that there may be more understanding in helping disciplines, such as social work, because of the emphasis on the use of self and social justice [31]. Other research has suggested that professional faculties (e.g., nursing, medicine, social work) may be riskier than other disciplines (e.g., sociology, gender studies, anthropology) because a record of harmful substance use could affect licensure [53]. Additional research is needed to explore the specific risks of FM addiction disclosure based on faculty type (e.g., professional vs. non-professional faculties).

### 5.5. Benefits of FM Disclosure

Despite the risks of FM addiction disclosure, an emerging body of literature highlights some of the unique opportunities connected to academics disclosing an addiction identity. For instance, two recent articles have argued that having professors disclose in undergraduate classrooms may be an effective way to combat stigma while also paving the way for students to seek help [1,9]. The benefits of addiction researcher disclosure have also been recently argued. Ross et al. (p. 2) suggest that not “coming out” as a drug researcher falls short of several qualitative methodological and ethical principles, including reflexivity and transparency. They write, “for researchers who value reflexivity, silence on this issue, therefore is bad science.” Along similar lines, in their recent commentary, Stull et al. [8] (p. 1) argue for more open disclosure of addiction researchers with addiction, as a means to “access insights and understandings that are unavailable to addiction researchers without similar experiences, and also reduce stigma, and ultimately bring greater awareness to the human-ness and heterogeneity of addiction.” 

Whether FMs decide to disclose an addiction and/or recovery identity is a deeply personal decision. Despite the call for more vulnerability, disclosure risks in the workplace are significant [27] and reinforced by other identity markers (e.g., race, gender, disability, etc.) [32]. For instance, although I (first author) went against my academic mentor’s advice and disclosed pre-tenure, I benefited from several privileges (e.g., being white, cis-gendered, having a supportive dean in a faculty of social work, and union support) that reduced disclosure risks. However, I was also aware that my other identities (e.g., being a female with visible and invisible disabilities) increased my risk of prejudice and discrimination [1,32]. 

Ultimately, it took me five years of negotiating my privacy boundaries [4] to reach the point where I could no longer conceal in the workplace. In the end, my decision to disclose came down to “strengthening my own personal recovery, combatting self and public stigma, helping build community, modelling authenticity and transparency in teaching and research roles, shifting university drinking culture, and providing a safer environment for others in less privileged positions (e.g., students) to disclose and/or seek help for addiction” [1] (p. 1). I also personally view addiction-recovery disclosure as a social justice issue—“recovering out loud” saves lives by providing hope to those who are still suffering in silence. 

## 6. Recommendations

### 6.1. Expanding Peer Support and Collegiate Recovery Programs for FMs

Key informants overwhelmingly recommended implementing dedicated addiction peer-support groups for FMs. Peer support involves individuals with lived experience of addiction and exists on a continuum: from informal volunteer mutual aid support systems, such as the 12-step model of AA, to formalized and employed paraprofessionals who require training and certification [54]. Livingston et al.’s [30] systematic review of stigma-reducing interventions for addiction showed that self-stigma could be reduced through peer-based support and acceptance. Peer support is currently considered best practice for addiction treatment, with outcomes that include reduction in emergency services usage and job loss, development of natural supports, encouragement toward a path of wellness and recovery, promotion of long-term symptom reduction, decreased risk of relapse, development of self-confidence and self-esteem, and efficacy in abstaining [55]. 

Peer support is a cornerstone of Collegiate Recovery Programs (CRPs), which have been implemented in 138 universities and colleges across the USA (two recently in Canada), and have shown to be effective in promoting recovery for students [56]. A CRP is a College or University provided, supportive environment within the campus culture that reinforces the decision to engage in a lifestyle of recovery from substance use and other addictive behaviors. It is designed to provide an educational opportunity alongside recovery support to ensure that students do not have to sacrifice one for the other [57]. Romo et al.’s [42] recent study exploring how college students in recovery manage the uncertainty of negotiating a recovery identity recommend an expansion of collegiate recovery programs, so that universities are not shying away from or viewing recovery as a “dirty little secret” [42] (p. 8). We suggest expanding CRP models to include FM specific programming. The services offered could be modeled off of CRP “best-practices” that have been identified for students, including dedicated university staff, built-in peer support and sober spaces, and drug-free events [56]. 

### 6.2. Adopting a Recovery-Friendly Workplace and Protective Policies

Traditional workplace responses to addiction focus largely on identifying and treating the “problem” of a limited number of people, which can reinforce stigma [27]. There have been recent calls for a “whole of workplace” approach [58] that considers shifting workplace culture and practices in order to reduce substance-related harms, promote recovery and wellness. Whole of workplace initiatives may involve employer/employee co-designing substance use policies, workplace education, and a more robust referral pathway to facilitate help seeking [58]. One example of a “whole of workplace” approach is the USA-based Recovery Friendly Workplace (RFW) initiative. Launched in 2018 by New Hampshire Governor Sununu, RFWs have been adopted by close to 280 organizations across the state [59]. Becoming a certified RFW requires ongoing training and programming consistent with recommendations from the addiction stigma literature [5], including addiction education, anti-stigma campaigns, resource referrals, and information to help create non-discriminatory substance use policies and procedures. When an organization becomes certified “Recovery-Friendly,” employees who experience problematic substance use can be assured that they will be met with compassion and support rather than punishment (e.g., immediate dismissal).

Even though addiction is a protected disability (e.g., under the American Disabilities Act [ADA], and the Accessible Canada Act), this protection is often disputed because people with addiction are viewed as being morally defective and thus less deserving of protection [60]. Legislation and policies need to be enforced in the workplace, so if FMs choose to disclose, regardless of where they are on their addiction journey, their employment will not be at risk. Relatedly, there is a need for increased awareness that addiction falls on a spectrum, and recovery is a process that often includes relapse [11]. 

Taken together, inspired by the findings of this study, the intervening literature, and consultations with CRPs and RFW leaders, I (first author) am leading an initiative working toward promoting recovery from addiction, building community, and combatting addiction stigma at my university. Relatedly, I started an Instagram page called @recoveringacademics, a community collective for academics in recovery and allies who are committed to addiction/recovery research and social justice. The page is profiling academics in recovery, with the aim to build community and reduce addiction stigma by celebrating recovery identities, as Dr. Wendy Dossett (personal communication, 11 April 2021) expressed:

*Academia exerts powerful and toxic identity orthodoxies which make addressing stigma within universities particularly challenging. I commend the @recoveringacademics initiative for legitimizing and celebrating a recovery identity, my identity, in what can sometimes be an inhospitable environment. Let’s make our universities recovery-friendly!*

## 7. Conclusions

This qualitative study explored 10 dean and 6 CMHPs encounters with FM addiction, how addiction stigma may affect FM (non) disclosure, and recommendations that may help reduce addiction stigma for FMs. Using a CPM framework, this study makes a unique contribution to the literature as it provides insights into how psychosocial factors including university norms (drinking, productivity culture), and identity markers (faculty type, gender) may affect addiction stigma and disclosure for FMs. Ultimately, there is a need to look beyond individual vulnerabilities and start examining organizational culture, policies, and procedures that help and/or hinder disclosure and help-seeking behaviors. Bolstering existing services available to FMs (e.g., Employee Assistant Programs [EAP]), challenging productivity culture and alcohol culture, sharing addiction-recovery narratives, and ensuring policy safeguards are in place, are promising ways forward to support addiction-recovery for FMs and the campus community at large.

## 8. Limitations and Future Research

While this study provided a glimpse into how deans and CMHPs experienced FM addiction disclosure, the findings need to be interpreted in view of their limitations. First, FMs themselves were not directly consulted about their addiction and disclosure experiences. Additional qualitative studies focusing on the direct experiences of FMs are needed, with a particular focus on the disclosure decision-making process. Additional quantitative research is also needed to evaluate future addiction anti-stigma interventions and determine the help-seeking behaviors for addiction and their prevalence among FMs in a larger sample across university settings.

Second, in recognizing an aim of qualitative research is transferability, not generalizability [44,61], and that the study was conducted in a single, large research-intensive Canadian university, the findings and implications may not be transferable to other contexts (e.g., smaller universities or colleges in different countries). Third, the small sample size did not allow for an in-depth exploration of how different identity markers (e.g., gender, ethnicity, rank) affected FM disclosure; this needs to be considered in future research. Relatedly, this study focused primarily on the dynamics of disclosing an alcohol addiction—a legal drug with a specific cultural context affecting disclosure motivation. The implications of disclosing illegal drugs would be very different and should be specifically considered in future research (e.g., see Ross et al. [31]). Fourth, the themes identified from the analysis are based on the views of 10 deans only. Interviewing all 16 deans would have provided a clearer picture of FM disclosure in this university.

Despite these limitations, exploring key informants’ encounters with FM addiction provides an enriched understanding of how university culture may affect addiction stigma and FM disclosure, while providing several practical recommendations and useful jumping off points for future research.

## Figures and Tables

**Table 1:** Interview Guide.

Discussion Area	Questions
(1) Encounters	Are you aware of or have you been approached by FM(s) for support with a substance-related addiction? Can you tell me about one or more experience(s) with a FM with substance-related addiction(s) (e.g., approached you for support, workload reduction, stress leave, etc.)? Have you had any concerns about FMs regarding substance-related addiction?
(2) Risks of self-disclosure (3) Recommendations	In your opinion, what are some of the risks for a FM to self-disclose a substance-related addiction? How do these risks differ from other professions, if at all? Do they differ for other members of the university community more broadly (e.g., staff, students)? In your opinion, what are some of the differences of self-disclosing and seeking help for a substance-related addiction compared to other mental health issues (e.g., anxiety, grief, depression)? Are you aware of any types of supports and resources for substance-related addiction on campus? If a FM approached you with a concern about substance-related addiction, what types of supports would you offer? What additional supports are needed for FMs with substance-related addiction (e.g., on and off campus)?

## Data Availability

Not applicable.
